# The correlation of age with chemotherapy-induced ovarian function failure in breast cancer patients

**DOI:** 10.18632/oncotarget.14532

**Published:** 2017-01-05

**Authors:** Ingeborg J.H. Vriens, Ashley J.R. De Bie, Maureen J.B. Aarts, Maaike de Boer, Irene E.G. van Hellemond, Joyce H.E. Roijen, Ron J.T. van Golde, Adri C. Voogd, Vivianne C.G. Tjan-Heijnen

**Affiliations:** ^1^ Department of Medical Oncology, GROW-School for Oncology and Developmental Biology, Maastricht University Medical Center, Maastricht, The Netherlands; ^2^ Department of Obstetrics and Gynaecology, Maastricht University Medical Center, Maastricht, The Netherlands

**Keywords:** chemotherapy induced ovarian function failure, breast cancer, chemotherapy, ovarian insufficiency, premenopausal patients

## Abstract

**Purpose:**

To assess the incidence of chemotherapy-induced ovarian function failure (COFF) based on estradiol and follicle stimulating hormone (FSH) monitoring in premenopausal women with hormone-receptor positive breast cancer treated with second and third generation (neo-)adjuvant chemotherapy.

**Results:**

We identified 115 eligible women. Two years after start of chemotherapy, COFF was significantly more often present in women ≥ 40 years (85.6%) as compared to women < 40 years (8.7%). Only age was significantly associated with COFF two years after start of chemotherapy (HR 12.26; 95% CI 5.21–28.86). In 50% of the patients, premenopausal hormone levels were the first or only evidence of ovarian function recovery (OFR).

**Materials and Methods:**

We included all premenopausal women with hormone-receptor positive breast cancer treated with anthracycline-based chemotherapy, with or without taxanes, in our university hospital in the Netherlands in the years 2005-2013. Patients were 3-monthly monitored for ovarian function. Cox proportional hazards model was used to determine the predictive impact of various parameters on the occurrence of COFF.

**Conclusions:**

After second- or third generation (neo-)adjuvant chemotherapy, COFF was still present in 8.7% of patients < 40 years after two years. FSH and estradiol monitoring may be relevant for those in whom ovarian function suppression is considered an additional effective endocrine treatment.

## INTRODUCTION

Approximately 30% of women diagnosed with breast cancer is premenopausal [1]. In the presence of prognostic unfavorable factors, (neo-)adjuvant systemic therapy is recommended in order to improve breast cancer survival. Systemic therapy may cause amenorrhea and premature menopause. The only clear predictor of chemotherapy-induced amenorrhea described in literature, is age [2, 3, 4, 5]. Older pre- and perimenopausal women (≥ 40 years) have greater odds to experience chemotherapy-induced amenorrhea than younger women (49–100% versus 10–71%) [2, 3, 4, 5]. Moreover, older (≥ 40 years) premenopausal women have a lower chance on ovarian function recovery (OFR) during follow-up than younger women (33% versus 68%, respectively) [2, 3, 4, 5].

During the last decades, a shift took place in the type of adjuvant chemotherapy in breast cancer patients. Currently, most patients receive an anthracycline and/or taxane-based regimen instead of an alkylating-based first-generation schedule (cyclophosphamide, methotrexate, 5-fluorouracil (CMF)), because of a higher efficacy in terms of breast cancer outcome [6]. Some studies report a lower incidence of chemotherapy-induced amenorrhea in patients receiving anthracycline-based regimes compared to those receiving CMF, attributable to lower dosages of cyclophosphamide [3]. Others suggest an increased incidence, indicating a gonadal toxic effect of anthracyclines [3, 7]. The impact of taxanes and endocrine treatment with tamoxifen or aromatase-inhibitors on the incidence of chemotherapy-induced amenorrhea is unclear [2, 3].

Ovarian function suppression can disrupt the quality of life in premenopausal women as a result of subsequent infertility and other menopausal related short- and long-term side effects [2, 8, 9, 10]. However, ovarian function suppression may also be beneficial since it may reduce the risk of breast cancer recurrence in patients with hormone-receptor positive breast cancer [11, 12, 13].

In this respect, it is important to realize that in literature premature ovarian function failure is frequently referred to as ‘chemotherapy-induced amenorrhea’, thereby being a clinical diagnosis. Since amenorrhea is a poor surrogate for ovarian function, we are one of the first to analyze follicle-stimulating hormone (FSH) and 17-beta estradiol (estradiol) blood levels during follow-up. Therefore we use ‘chemotherapy-induced ovarian function failure (COFF)’ as definition. To avoid misunderstanding we only use ‘chemotherapy-induced amenorrhea’ in our paper when referring to the literature, because most of the literature did not take changes of hormonal levels into account.

The aim of this retrospective study was to evaluate the incidence of COFF and the rate of recovery from COFF in a cohort of premenopausal and perimenopausal women with hormone-receptor positive early stage breast cancer, treated with adjuvant anthracycline-based chemotherapy with or without taxanes.

## RESULTS

### Patient characteristics

We identified 148 premenopausal patients with early stage hormone-receptor positive breast cancer, who did not undergo ovarian ablation or immediate treatment with gonadotropin-releasing hormone (GnRH). In 6 patients, hormone levels were not measured at follow-up. 21 patients were considered unable to be evaluated for the study end points, because of follow-up with FSH and estradiol levels < 2 years. Another 6 patients, developed metastatic disease during 2-year follow-up. Therefore, 115 patients were eligible for the study.

The majority of patients were 40 years or older (78.3%). Patients who developed OFR after COFF had a mean age of 37.5 (SD 6.0) years at time of diagnosis. Patients who did not develop OFR had a mean age of 47.4 (SD 3.9) years at time of diagnosis. Delivered chemotherapy consisted of six cycles of FEC_100_ (5-fluorouracil, epirubicin, cyclophosphamide; *n* = 19, 16.5%), 4–4 cycles AC-T (4 cycles of adriamycine and cyclophosphamide followed by 4 cycles of docetaxel; *n* = 3, 2.6%) or six cycles TAC (combination of docetaxel, adriamycine and cyclophosphamide; *n* = 93, 80.9%). All but six of 115 patients with a hormone-receptor positive tumor received adjuvant endocrine therapy, of whom 99 started with tamoxifen and 10 with aromatase inhibitor immediately after chemotherapy. The median number of FSH/estradiol measurements was 4 (range 1–8).

In 73.5% of the patients with COFF, premenopausal hormone levels were the first evidence of OFR (Table 1). In the remaining 26.4%, resumption of menses was the first sign of OFR; in all except one of these patients, FSH and estradiol levels were used to confirm the OFR after COFF. The second-last FSH and estradiol levels of patients who had OFR after COFF were still clearly in postmenopausal range.

**Table 1:** Evidence for OFR in patients with OFR after COFF

	No. (total 34)	%
Premenopausal hormone levels first, later menses	8	23.5
Premenopausal hormone levels only, no menses	17	50
Menses first, confirmed by hormone levels	8	23.5
Menses only, no hormone levels (not measured)	1	2.9

### Occurrence of COFF

COFF was initially present in 113 (98.3%) of 115 assessable patients; that is, in all (*n* = 90) patients of ≥ 40 years versus in 23 of 25 (92.0%) of patients < 40 years of age (*P* = < 0.001). At a minimum follow-up of two years, the ovarian function of 34 of 113 women had recovered. At a follow-up of 2 years, 8.7% of the patients < 40 years and 85.6% of the patients ≥ 40 years still had COFF.

### Incidence of and parameters associated with OFR after COFF

A multivariate logistic regression model was used to analyze the impact of age, family history, chemotherapy, and endocrine therapy on COFF. The results demonstrated that younger age was independently related to an increased risk of OFR after COFF, with a hazard ratio (HR) of 12.26 (95% CI 5.21–28.86, *P* = < 0.001) (Table 2). The other factors, family history, type of chemotherapy and of endocrine therapy were not significantly related with OFR.

**Table 2:** Univariate and multivariate analysis of associations between prognostic factors of OFR after COFF and COFF two years after chemotherapy

Patient characteristics		OFR after COFF	COFF two years after chemotherapy	Univariate analysis		Multivariate analysis	
		*N*	*N*	Hazard ratio	95% CI	Hazard ratio	95% CI
Age	< 40 years	21	2	11.83	5.77–24.27	12.26	5.21–28.86
	≥ 40 years	13	77				
Family history	Positive	10	29	0.73	0.35–1.52	0.57	0.26 – 1.26
	Negative	24	50				
Taxane-based chemotherapy	Yes	32	64	3.14	0.75–13.12	1.06	0.22–5.02
	No	2	15				
Endocrine therapy							
Tamoxifen only	Yes	29	47	1 (Ref)		1 (Ref)	
	No	5	32				
Aromatase inhibitor only	Yes	2	10	9.27	1.26–68.07	3.16	0.38–26.20
	No	32	69				
Sequential	Yes	1	20	3.65	0.33–40.03	3.17	0.29–35.24
	No	33	59				

COFF = Chemotherapy-induced ovarian function failure. OFR = Ovarian function recovery. CI = Confidence interval.

### Time to recovery of ovarian function

**Figure 1:**
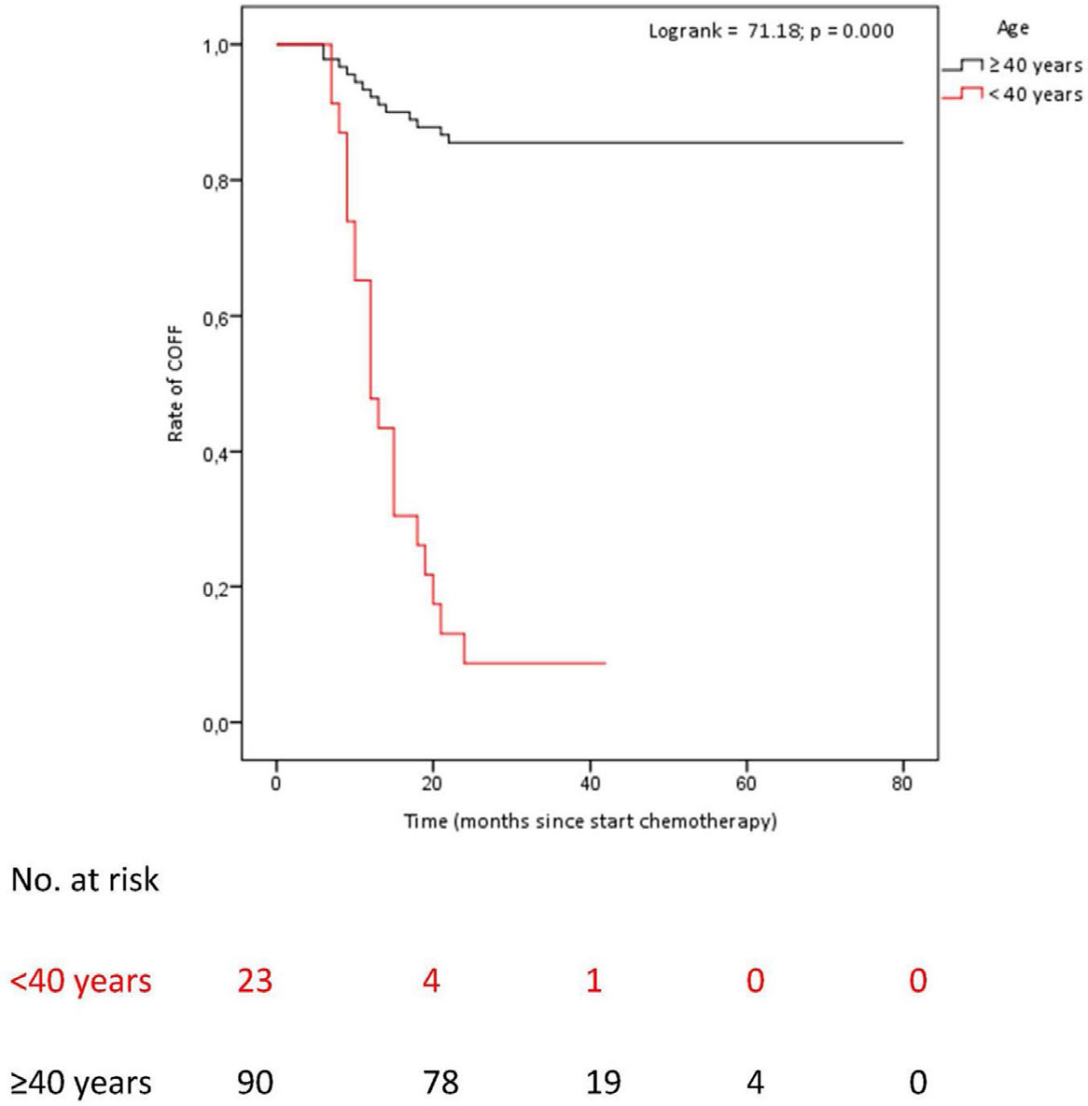
Time untill OFR after COFF in premenopausal patients who had recieved (neo)adjuvant chemotherapy COFF = Chemotherapy-induced ovarian function failure. OFR = Ovarian function recovery. Patients < 40 years of age: COFF in 92% of whom 8.7% COFF 2 years after chemotherapy. Patients ≥ 40 years of age: COFF in 100%, of whom 85.6% COFF 2 years after chemotherapy.

Of the women, whose ovarian function after COFF had recovered after a minimum follow-up of 2 years (*n* = 34), 59.4% recovered within one year after start chemotherapy (Figure 1). Of patients < 40 years of age: 8.7% had no recovery of ovarian function after COFF 2 years after chemotherapy. Of patients ≥ 40 years of age: 85.6% had no recovery of ovarian function after COFF 2 years after chemotherapy (Figure 1).

### FSH and estradiol levels during follow-up

**Figure 2:**
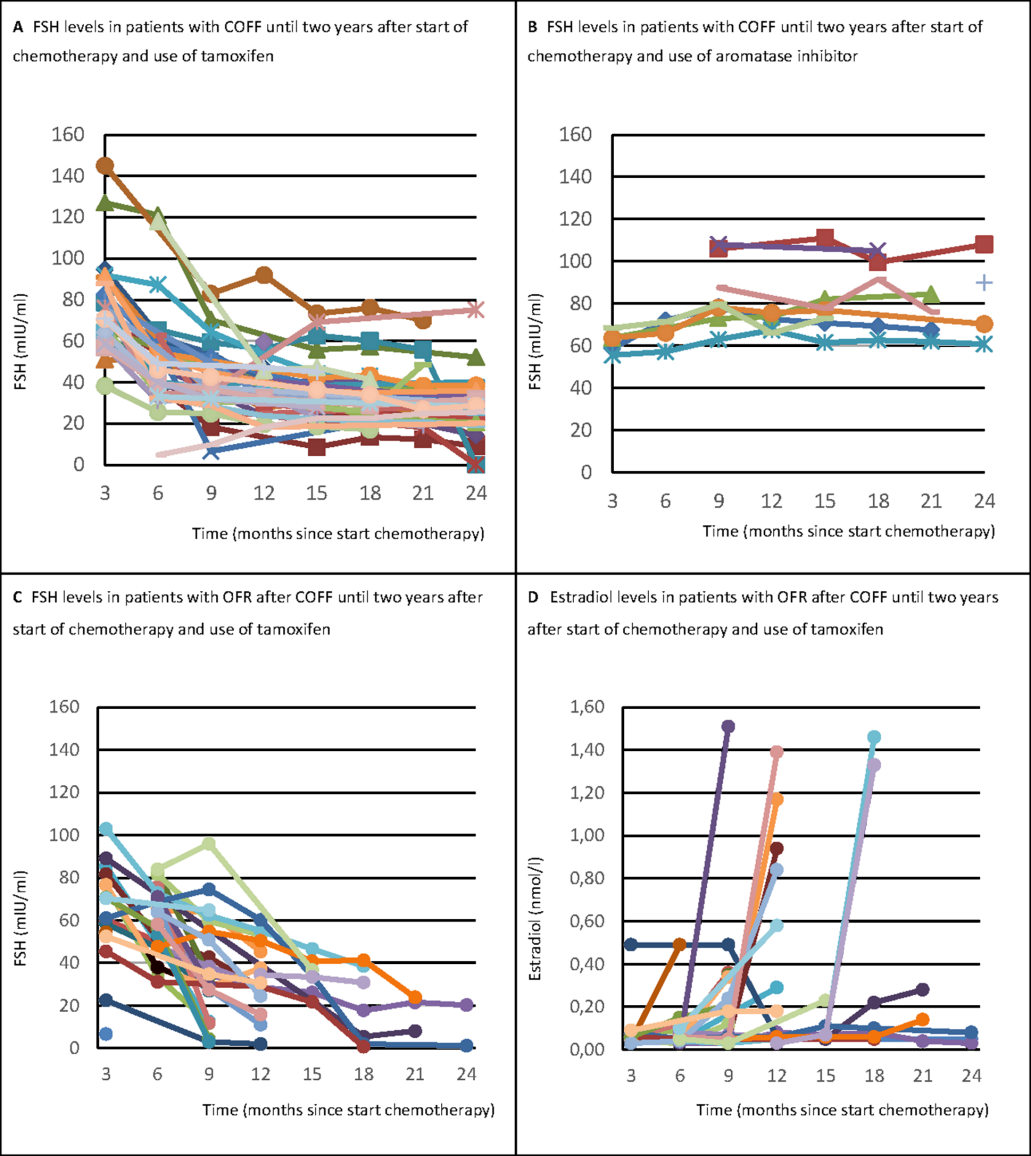
FSH and estradiol levels in patients with OFR after COFF two years after chemotherapy, treated with tamoxifen or aromatase inhibitors COFF = Chemotherapy-induced ovarian function failure. OFR = Ovarian function recovery. FSH = Follicle stimulating hormone. Panel A shows that the initially highly increased FSH levels rapidly declined during treatment with tamoxifen, whereas Panel B shows that in patients receiving aromatase inhibitors FSH levels continued to be high. Further, it is shown that when the ovarian function recovered after initial COFF in patients treated with tamoxifen, both FSH (Panel C) and estradiol levels (Panel D) rapidly normalized to premenopausal levels at the same time.

In patients who after two years still had COFF, the initially highly increased FSH levels rapidly declined during treatment with tamoxifen, whereas in patients receiving aromatase inhibitors FSH levels continued to be high (Figure 2A and 2B).

When ovarian function recovered after initial COFF, both FSH and estradiol levels normalized rapidly to premenopausal levels at the same time (Figure 2C and 2D).

## DISCUSSION

We performed a single-institution chart review to determine the rate of premature menopause in young patients with hormone-receptor positive breast cancer largely treated with third-generation (neo-) adjuvant chemotherapy. Of note, in contrast to other studies, we did not only record occurrence of amenorrhea, but we also monitored ovarian function by regularly measuring FSH and estradiol levels. We observed, that both young and older premenopausal breast cancer patients were frequently confronted with the occurrence of COFF (97.7%). However, in the majority of young women, ovarian function recovered in the months-few years thereafter. Two years after start of chemotherapy, COFF was significantly more often present in women ≥ 40 years (85.6%) as compared to women < 40 years (8.7%). Younger age was independently related to an increased risk of OFR after COFF with a HR of 12.26 (95% CI 5.21–28.86). Moreover, we noticed that in half of patients, premenopausal hormone levels were the first or only evidence of OFR.

In literature, the incidence of chemotherapy-induced amenorrhea ranges from 16–85% in women younger than 40 years old compared to 60–98% in older premenopausal women [2, 3, 4, 5]. These wide ranges might be explained due to the heterogeneity of the definitions and follow-up duration, which varies in literature from absence of menstruation for 6 months to 12 months after chemotherapy [2, 3, 4, 5, 14]. More importantly, previous studies on chemotherapy-induced amenorrhea only considered presence or absence of menstrual cycles [2–5, 7, 9, 11, 14–18]. We showed, however, that in nearly one third of patients amenorrhea was still present despite OFR as documented by rise in estradiol levels. Hence, a clinical definition of chemotherapy-induced menopause solely based on absence of menstrual cycles may hugely overestimate permanent ovarian function suppression. Another explanation for these wide ranges is the different regimens of chemotherapy used as treatment. The kind of regimen used is a predictive factor for the development of chemotherapy-induced amenorrhea; especially cyclophosphamide-containing chemotherapy is associated with chemotherapy-induced amenorrhea [2, 3, 17].

The strongest predictor for experiencing COFF two years after chemotherapy is older age (≥ 40 years). Remarkable in our series was that in another 8% of all patients with OFR after COFF, the ovarian function still recovered after two years. In the patients with OFR after COFF, the second-last FSH and estradiol levels were still clearly in postmenopausal range, so recovery of ovarian function can discretely appear within the five years of endocrine treatment. This is a dangerous phenomenon, as it potentially reduces anticancer activity and even pregnancy can occur, especially in patients receiving aromatase inhibition [19, 20].

Another remarkable observation was the trend shown in the figures displaying levels of FSH during COFF. The steep decline of FSH levels caused by tamoxifen (Figure 2A and 2C) was already described in literature [21, 22, 23]. The hypothesis for this reduction are estrogen-like actions of tamoxifen on gonadotropin secretion. This induces an increased negative feedback on the hypothalamic-ovarian axis and decreases FSH levels resulting in suppression of the ovaries [4, 18, 22]. This is also hypothesized by large studies to explain the increased incidence of chemotherapy-induced amenorrhea seen in patients treated with tamoxifen [2, 11, 14]. Our series also showed an effect of treatment with tamoxifen on the incidence of COFF.

We noticed that hormone levels abruptly changed to premenopausal levels when the ovarian function recovered. Therefore, FSH and estradiol are better markers to diagnose OFR than to predict recovery of ovarian function. A possible explanation could be that the hypothalamic-ovarian axis recovers and may maintain itself as soon as one follicle is able to fully mature. Although FSH can be used as a marker to diagnose OFR, physicians should be cautious with interpreting FSH levels alone [23]. Our study shows that tamoxifen can decrease FSH levels to very low levels (levels that can also be seen in premenopausal patients), while estradiol levels are still in a postmenopausal state. In these cases it is recommended to take the levels of estradiol always into consideration before diagnosing OFR.

Aromatase inhibitors promote ovulation in premenopausal women with fertility problems [24]. Therefore concern rises when premenopausal patients with COFF are treated with aromatase inhibitors [25]. Our series could not confirm any significant association between the rate or time to OFR and any endocrine treatment. The most probable explanation is that only few patients were treated with aromatase inhibitors. Henry et al. showed in a prospective clinical trial of pre- en perimenopausal women ≥ 40 years (range 40–51 years) with biochemically confirmed COFF, that about one-quarter (13 out of 45) of women recovered ovarian function during aromatase inhibitors – therapy [26]. Many oncologists prescribe aromatase inhibitors treatment to women older than 40 years who have experienced chemotherapy-induced amenorrhea. However, as shown in the current study, amenorrhea at the end of chemotherapy is not an accurate guide to the underlying ovarian function. Aromatase inhibitors are pharmacologically ineffective in women with functioning ovaries. They may even promote the recovery of ovarian function in younger women, antagonizing the anticancer efficacy and therefore aromatase inhibitors are contra-indicated in women with functioning ovaries [26]. In the Austrian Breast and Colorectal Cancer Study Group–12 (ABCSG-12) trial, an unfavorable trend was found for the use of anastrozole as compared to tamoxifen in combination with ovarian function suppression with respect to overall survival [27]. The SOFT trial demonstrated a significant improvement in overall survival and a trend to better disease free survival in women treated with chemotherapy and with tamoxifen combined with ovarian function suppression. This effect is highest in young women with a high risk of recurrence [12]. Therefore monitoring of ovarian function may become extremely important, requiring standardization of FSH and estradiol essays. Using the wrong assays has led to inaccurate data in some recently published studies [28].

So in general, for young women who have a hormone-receptor positive breast cancer, OFR after COFF may be unwanted because of reduced effectiveness of adjuvant endocrine treatment [12]. But, in young breast cancer patients with an unfullfilled disease to have children, OFR after COFF can be extremely desirable. Though it is important to understand that OFR after COFF is not directly equal to fertility, our findings of a low COFF rate two years after chemotherapy may be reassuring for young women still having an unfulfilled desire to have children [29–31]. A Swedish study showed that most cancer survivors who had a pretreatment desire for children, still wanted children 3–7 years after treatment [32]. In a US study, the majority of women concerned about fertility at cancer diagnosis did not make use of fertility preservation techniques [29]. Recently another US study showed that patient satisfaction improved with information received about fertility, demonstrating the potential for fertility programs in cancer centers [33]. A meta-analysis and two recent randomized controlled trials showed that temporary ovarian function suppression induced by gonadotropin-releasing hormone (GnRH) agonists during chemotherapy may reduce the risk of chemotherapy-induced amenorrhea [34, 35, 36]. However, interpretation is complicated because the definition of ovarian function failure varied among the trials, many data were missing, first-generation chemotherapy regimens were used and patients ≥ 40 years were included. More importantly, considering the low 2-year COFF rate if below 40 years at breast cancer diagnosis, one may also question whether use of GnRH agonists is of clinical relevance. The use of GnRH agonists to retain fertility may be more limited. Only 3% of patients with ER-positive tumors and only 14% of those with ER-negative tumors carried a pregnancy to term.

Limitations of this study are inherent to its retrospective design. Biases and inaccuracies may occur as a result of the reliance on the interpretations of different physicians and integrity of information in the medical records. Some patients (< 40 years), for example, received preventive GnRH-agonists or oophorectomies, even though they experienced COFF. Therefore these patients were excluded, which reduced the population of younger women in our study. Moreover, in our study the follow-up duration was still relatively short. This implies that the number of patients with OFR may increase even further over time.

We conclude that a younger age (< 40 years) was significantly associated with a higher rate of OFR after COFF. This seems reassuring for those with a desire to have children. As in a significant proportion of patients FSH and estradiol levels were the first sign of OFR, close monitoring of ovarian function is required if ovarian function suppression is considered an additional effective endocrine treatment. In clinical practice we suggest to routinely check FSH and estradiol levels in patients experiencing COFF for at least 5 years, because in at least 8% of the patients with OFR after COFF, the ovarian function recovered even after two years. We recommend not to use aromatase inhibitors as single endocrine treatment in young patients with COFF. The results from our observational study can be used to inform young breast cancer patients (< 40 years) about the risks of COFF. To avoid incongruent definitions we strongly argue to use only one definition for chemotherapy-induced ovarian function failure (COFF) based on serial FSH and estradiol levels in future trials.

## MATERIALS AND METHODS

### Study design

This study is a retrospective cohort study. We retrieved data on patient and tumor characteristics and on delivered systemic treatment from the medical records of patients < 55 years. We collected the available information on menstrual cycle and on the levels of FSH and estradiol during the follow-up outpatient visits. In our hospital, the policy for patients with COFF and hormone-receptor positive breast cancer is to routinely monitor ovarian function by 3-monthly FSH and estradiol blood levels. Serum estradiol is measured by a direct immunoassay with high sensitivity in the lower ranges. Because of the retrospective design of our study, the study did not fall under the scope of the act of medical scientific research and did not have to be reviewed by an accredited research ethics committee.

### Patients

All pre- and perimenopausal women with hormone-receptor positive stage I–III breast cancer who received (neo-) adjuvant chemotherapy in the Maastricht University Medical Centre between 2005–2013 were included. Exclusion criteria were hormone-receptor negative breast cancer, a follow-up with FSH and estradiol blood levels of less than 24 months, no monitoring of ovarian functioning, metastatic disease at diagnosis or during 2-year follow-up, a medical history of other malignancies and previous treatment with chemotherapy. Also patients who have had a (prophylactic) surgical ovarian ablation or who received gonadotropin-releasing hormone (GnRH) agonist immediately after the end of chemotherapy were excluded.

### Definitions

Premenopausal status was defined as having regular menses in the last year before the start of (neo-) adjuvant chemotherapy. Perimenopausal status was defined as having irregular menses and at least one menstrual cycle within six months before the start of the chemotherapy. Patients who have had hysterectomy without bilateral oophorectomy were eligible for inclusion only if serum estradiol and FSH were consistent with pre-menopausal status within 3 months before chemotherapy initiation. Patients < 55 years using oral contraceptives at diagnosis were classified as premenopausal.

To meet the definition of COFF, postmenopausal ovarian function according to our institutional standards (FSH > 21.9 mIU/ml and estradiol < 0.11 nmol/l) had to be present after chemotherapy. When we could not establish the exact date of the last menstruation during chemotherapy, the date of the second chemotherapy cycle was used.

We defined recovery of ovarian function as resumption of menses or when levels of biochemical markers did not correlate anymore with the levels seen in a postmenopausal state according to our institutional standards (FSH > 21.9 mIU/ml and estradiol < 0.11 nmol/l) after previously confirmed COFF.

### Study endpoints

The primary aim of our study was to determine the rate of OFR after COFF in pre- and perimenopausal women with hormone-receptor positive breast cancer, 2 years after having started anthracycline-based (neo-) adjuvant chemotherapy with or without taxanes in a real-life setting. Secondary aims were to assess possible associations between rate of OFR and age or other factors, time to OFR and levels of FSH and estradiol in patients receiving tamoxifen or aromatase inhibitors.

### Statistical analysis

All statistical analyses were performed with SPSS 22.0. The time and rate of resuming ovarian function was estimated with the Kaplan-Meier method, comparing the curves with a log rank test. A multivariate Cox analysis was used to assess the independent impact of the relevant variables on predicting the incidence of COFF and OFR. These variables included age, family history, taxane-based chemotherapy and endocrine therapy. In all statistical analyses a *P*-value of < 0.05 was considered as statistically significant.

## References

[1] Torino F, Barnabei A, De Vecchis L, Sini V, Schittulli F, Marchetti P, Corsello SM (2014). Chemotherapy-induced ovarian toxicity in patients affected bij endocrine-responsive early breast cancer. Critical Reviews in Oncology/Hematology.

[2] Walshe JM, Denduluri N, Swain SM (2006). Amenorrhea in premenopausal women after adjuvant chemotherapy for breast cancer. J Clin Oncol.

[3] Tham YL, Sexton K, Weiss H, Elledge R, Friedman LC, Kramer R (2007). The rates of chemotherapy-induced amenorrhea in patients treated with adjuvant doxorubicin and Cyclophosphamide followed by a taxane. Am J Clin Oncol.

[4] Yung M, Shin HJ, Rha SY, Jeung HC, Hong S, Moon YW, Kim HS, Oh KJ, Yang WI, Roh JK, Chung HC (2010). The clinical outcome of chemotherapy-induced amenorrhea in premenopausal young patients with breast cancer with long-term follow-up. Ann Surg Oncol.

[5] Okanami Y, Ito Y, Watanabe C, Iijima K, Iwase T, Tokudome N, Takahashi S, Hatake K (2011). Incidence of chemotherapy-induced amenorrhea in premenopausal patients with breast cancer following adjuvant anthracycline and taxane. Breast Cancer.

[6] Breast Early, Cancer Trialists’ Collaborative Group (EBCTCG) (2012). Comparisons between different polychemotherapy regimens for early breast cancer: meta-analyses of long-term outcome among 100 000 women in 123 randomised trials. Lancet.

[7] Parulekar WR, Day AG, Ottaway JA, Shepherd LE, Trudeau ME, Bramwell V, Levine L, Pritchard KI (2005). Incidence and prognostic impact of amenorrhea during adjuvant therapy in high-risk premenopausal breast cancer: analysis of a National Cancer Institute of Canada Clinical Trials Group Study—NCIC CTG MA.5. J Clin Oncol.

[8] Maltaris T, Weigel M, Mueller A, Schmidt M, Seufert R, Fischl F, Koelbl H, Dittrich R (2008). Cancer and fertility preservation: fertility preservation in breast cancer patients. Breast Cancer Res.

[9] Petrek JA, Naughton MJ, Case LD, Paskett ED, Naftalis EZ, Singletary SE, Sukumvanich P (2006). Incidence, time course, and determinants of menstrual bleeding after breast cancer treatment: a prospective study. J Clin Oncol.

[10] Gerber B, von Minckwitz G, Stehle H, Reimer T, Felberbaum F, Maass N, Fischer D, Sommer HL, Conrad B, Ortmann O, Fehm T, Rezai M, Meyta K (2011). Luteinizing Hormone-Releasing Hormone Agonist on Ovarian Function After Modern Adjuvant Breast Cancer Chemotherapy: The GBG 37 ZORO Study. J Clin Oncol.

[11] Swain SM, Land SR, Ritter MW, Costantino JP, Cecchini RS, Mamounas EP, Wolmark N, Ganz PA (2009). Amenorrhea in premenopausal women on the doxorubicin-and-Cyclophosphamide followed-by-docetaxel arm of NSABP B-30 trial. Breast Cancer Res Treat.

[12] Francis PA, Regan MM, Fleming GF, Láng I, Ciruelos E, Bellet M, Bonnefoi HR, Climent MA, GA Da Prada, Burstein HJ, Martino S, Davidson NE, Geyer CD (2015). Adjuvant Ovarian Suppression in Premenopausal Breast Cancer. N Eng J Med.

[13] Pagani O, Regan MM, Walley BA, Fleming FD, Colleoni M, Láng I, Gomez HL, Tondini C, Burstein HJ, Perez EA, Ciruelos E, Stearns V, Bonnefoi HR (2014). Adjuvant exemestane with ovarian suppression in premenopausal breast cancer. N Engl J Med.

[14] Colleoni M, Gelber S, Goldhirsch A, Aebi S, Castiglione-Gertsch M, Price KN, Coates AS, Gelber RD (2006). Tamoxifen after adjuvant chemotherapy for premenopausal women with lymph node-positive breast cancer: International Breast Cancer Study Group Trial 13–93. J Clin Oncol.

[15] Bines J, Oleske DM, Cobleigh MA (1996). Ovarian function in premenopausal women treated with adjuvant chemotherapy for breast cancer. J Clin Oncol.

[16] Han HS, Ro J, Lee KS, Nam BH, Seo JA, Lee DH, Lee H, Lee ES, Kang HS, Kim SW (2009). Analysis of chemotherapy-induced amenorrhea rates by three different anthracycline and taxane containing regimens for early breast cancer. Breast Cancer Res Treat.

[17] Najafi S, Djavid GE, Mehrdad N, Rajaii E, Alavi N, Olfatbakhsh A, Najafi M, Bahrami A, Heidari K (2011). Taxane-based regimens as a risk factor for chemotherapy-induced amenorrhea. Menopause.

[18] Amir E, Freedman O, Allen E, Colgan T, Clemons M (2010). Defining ovarian failure in amenorrheic young breast cancer patients. Breast.

[19] Breast Early, Cancer Trialists’ Collaborative Group (2005). Effects of chemotherapy and hormonal therapy for early breast cancer on recurrence and 15-year survival: an overview of the randomised trials. Lancet.

[20] Ward JH (2010). Duration of adjuvant endocrine therapy of breast cancer: how much is enough?. Curr Opin Obstet Gynecol.

[21] Rose DP, Davis TE (1980). Effects of adjuvant chemohormonal therapy on the ovarian and adrenal function of breast cancer patients. Cancer Res.

[22] Rossi E, Morabito A, Di Rella F, Esposito G, Gravina A, Labonia V, Landi G, Nuzzo F, Pacilio C, De Maio E, Di Maio M, Piccirillo MC, De Feo G, Gallo C, Perrone F, de Matteis A (2009). Endocrine effects of adjuvant letrozole compared with tamoxifen in hormone-responsive postmenopausal patients with early breast cancer: the HOBOE trial. J Clin Oncol.

[23] Henry NL, Xia R, Schott AF, McConnell D, Banerjee M, Hayes DF (2014). Prediction of Postchemotherapy Ovarian Function Using Markers of Ovarian Reserve. The Oncologist.

[24] Legro RS, Brzyski RG, Diamond MP, Coutifaris C, Schlaff WD, Casson P, Christman GM, Huang H, Yan Q, Alvero R, Haisenleder DJ, Barnhart KT, Bates GW (2014). Letrozole versus clomiphene for infertility in the Polycystic Ovary Syndrome. N Eng J Med.

[25] Smith IE, Dowsett M, Yap Y-S, Walsh G, Lønning PE, Santen RJ, Hayes D (2006). Adjuvant aromatase Inhibitors for Early Breast Cancer After Chemotherapy-Induced Amenorrhoea: Caution and Suggested Guidelines. J Clin Oncol.

[26] Henry NL, Xia R, Banerjee M, Gersch C, McConnell D, Giacheri D, Schott AF, Pearlman M, Stearn V, Partridge AH, Hayes DF (2013). Predictors of recovery of ovarian function during aromatase inhibitor therapy. Annals of Oncology.

[27] Gnant M, Mlineritsch B, Stoeger H, Luschin-Ebengreuth G, Knauer M, Moik M, Jakesz R, Seifert M, Taucher S, Bjelic-Radisic V, Balic M, Eidtmann H, Eiermann W (2015). Zoledronic acid combined with adjuvant endocrine therapy of tamoxifen versus anastrozol plus ovarian function suppression in premenopausal early breast cancer: final analysis of the Austrian Breast and Colorectal Cancer Study Group Trial 12. Annals of Oncology.

[28] Folkerd EJ, Lønning PE, Dowsett M (2014). Interpreting Plasma Estrogen Levels in Breast Cancer: Caution Needed. J Clin Oncol.

[29] Ruddy KJ, Gelber SI, Tamimi RM, Ginsburg ES, Schapira L, Come SE, Borges VF, Meyer ME, Partrigde AH (2014). Prospective study of fertility concerns and preservation strategies in young women with breast cancer. J Clin Oncol.

[30] Partridge AH, Ruddy KJ (2007). Fertility and adjuvant treatment in young women with breast cancer. The Breast.

[31] Jeruss JS, Woodruff TK (2009). Preservation of fertility in patients with cancer. N Eng J Med.

[32] Armuand GM, Wettergren L, Rodriguez-Wallberg KA, Lampic C (2014). Desire for children, difficulties achieving a pregnancy, and infertility distress 3 to 7 years after cancer diagnosis. Support Care Cancer.

[33] Kelvin JF, Thom B, Benedict C, Carter J, Corcoran S, Dickler MN, Goodman KA, Margolies A, Matasar MJ, Noy A, Goldfarb SB (2016). Cancer and fertility program improves patient satisfaction with information recieved. J. Clin Oncol.

[34] L Del Mastro, Ceppi M, Poggio F, Bighin C, Peccatori F, Demeestere I, Levaggi A, Giraudi S, Lambertini M, D’Alonzo A, Canavese G, Pronzato P, Bruzzi P (2014). Gonadotropin-releasing hormone analogues for the prevention of chemotherapy-induced premature ovarian failure in cancer women: Systematic review and meta-analysis of randomized trials. Cancer Treatment Reviews.

[35] Moore HCF, Unger JM, Philips K-A, Boyle F, Hitre E, Porter D, Francis PA, Goldstein LJ, Gomez HL, Vallejos CS, Partridge AH, Shaker RD, Garcia AA (2015). Goserelin for Ovarian Protection during Breast-Cancer Adjuvant Chemotherapy. N Eng J Med.

[36] Lambertini M, Boni L, Michelotti A, Gamucci T, Scotto T, Gori S, Giordano M, Garonne O, Levaggi A, Poggio F, Giraudi S, Bighin C, Vecchio C (2015). Ovarian suppression with triptorelin during adjuvant breast cancer chemotherapy and long-term ovarian function, pregnancies, and disease-free survival. A Randomized Clinical Trial. JAMA.

